# CAREGIVER PREFERRED STRUCTURE FOR A TELEHEALTH-BASED INTERVENTION ONE-TO-ONE VERSUS GROUP

**DOI:** 10.1093/geroni/igz038.3165

**Published:** 2019-11-08

**Authors:** Sabrina J Shofner, Glenise McKenzie, David LaFazia, Loriann McNeill, Natasha Spoden, Nora Mattek, Linda Teri, Allison Lindauer

**Affiliations:** 1 OHSU, Portland, Oregon, United States; 2 Oregon Health & Science University, Portland, Oregon, United States; 3 University of Washington, Seattle, Washington, United States; 4 Multnomah County, Portland, Oregon, United States; 7 Oregon Alzheimer’s Disease Research Center, Portland, Oregon, United States

## Abstract

Caring for a person with Alzheimer’s disease and related dementias (ADRD) can be stressful. Support programs are available for caregivers, but distance and cost present barriers to participation. Our mixed-methods study explored consumer acceptability and preliminary efficacy of a telehealth-based caregiver intervention, Tele-STAR. Caregivers in Tele-STAR met one-to-one with a consultant over eight weeks, via video conferencing, to address behavioral symptoms of dementia. We measured the effect of the intervention on caregiver reactivity to behavioral symptoms. Focus groups were used to assess acceptability. The literature suggests that stressed caregivers drop out of support programs. Thus, in the focus groups we asked about preference for intervention mode (one-to-one versus group). We hypothesized that more stressed caregivers would prefer the one-to-one mode. Data were analyzed using paired t-tests for the quantitative data, and a phenomenological lens for the qualitative data. Of the thirteen enrolled caregivers, twelve completed the study. Significant improvement was found in caregiver reactivity (p=0.001). Twelve caregivers participated in focus groups, in which they reported that the intervention helpful and they valued the therapeutic relationships with the consultants. Few had difficulty with the technological interface. Most liked the one-to-one mode of the intervention, but were open to a hypothetical group-based option. However, more stressed caregivers preferred a one-to-one intervention over a group intervention (p=0.06). While one-to-one interventions tend to be preferred by caregivers, they are expensive. Our findings suggest that one-to-one telehealth-based interventions should be reserved for more stressed caregivers who need intensive support with managing behavioral symptoms of dementia.

